# Supplementary material for: Diffuse neural coupling mediates complex network dynamics through the formation of quasi-critical brain states
Source: Nat Commun. 2020 Dec 10;11:6337. doi: 10.1038/s41467-020-19716-7 (PMC7729877; doi:10.1038/s41467-020-19716-7)
Supplement: Supplementary file 1 — Supplementary Information [file 41467_2020_19716_MOESM1_ESM.pdf]

Supplementary material

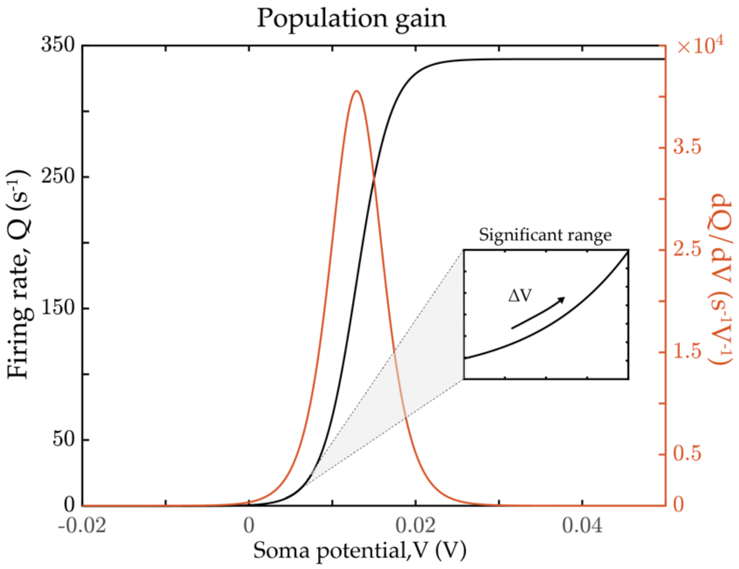

Figure S1 –Population firing rate gain. (left: black**)** sigmoidal mapping of firing rate to soma potential; (right: orange) first derivative of the sigmoid function. The inset shows the range of postsynaptic potentials significant for the steady-state population firing rates as a function of induced postsynaptic potential. Network coupling in this model is facilitated by connecting cortical excitatory populations between each node. This means that as diffuse coupling increases, the network-induced positive postsynaptic potential generated increases in the receiving nodes cortical population. The effect of this increase can be seen in Fig. S2(a) where a sweep of constant postsynaptic potential shows the deformation of steady state firing rate attractors. At ~0.12 mV, the low attractor is lost through a saddle-node bifurcation leaving only the high firing attractor. Notably, the relative difference of the thalamic reticular nuclei (TRN) and specific relay nuclei (SRN) firing rate attractors is inverted either side of the bifurcation. The simulated time series of cortical firing rates, given in Fig. S2 (b), can then be understood as a 'smearing' of the results in Fig. S2(a).

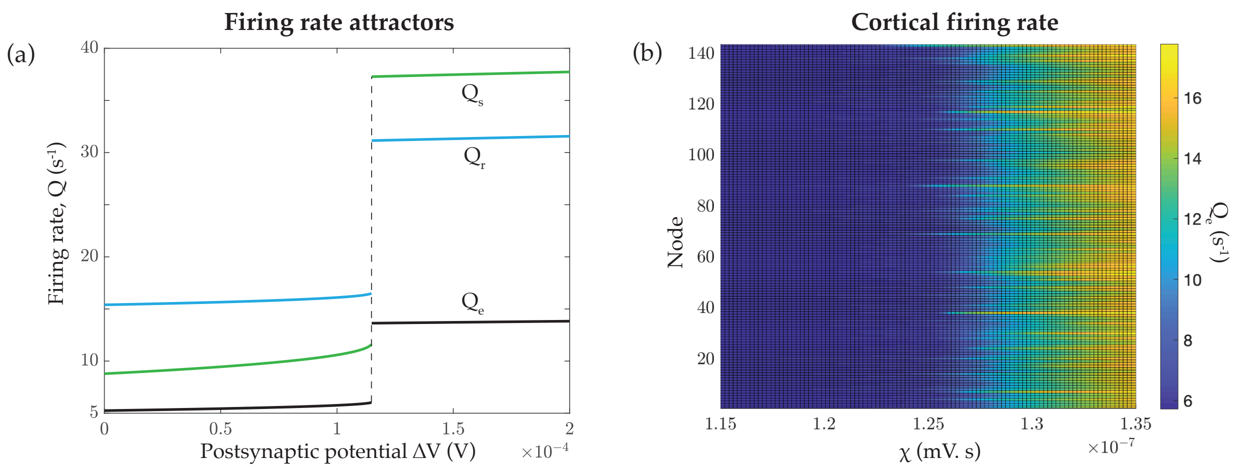

Figure S2– Corticothalamic firing rate attractors across the critical point. (a) Steady-state population firing rates as a function of induced postsynaptic potential. (Qe – cortical excitatory nuclei, Qs – thalamic specific relay nuclei, Qr – thalamic reticular nucleus). (b) Simulated mean cortical firing rate for each node as a function of diffuse coupling.

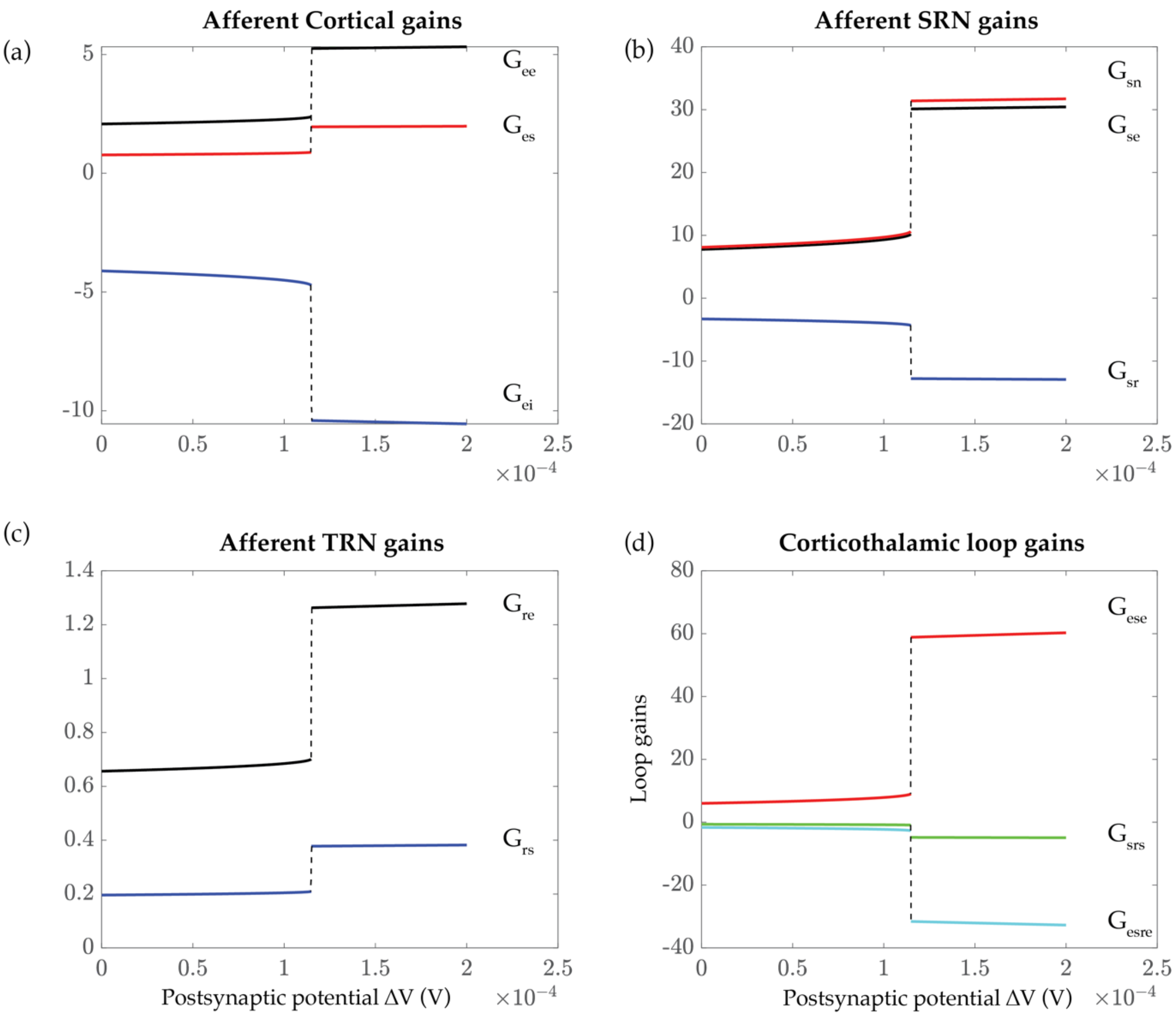

Figure S3 – Corticothalamic steady state gains across the critical point. (a) Excitatory cortical input gains from the excitatory (G_ee), and inhibitory (G_ei) cortex, and the SRN (G_es). (b) SRN thalamic input gains from the cortex (G_se), TRN (G_sr), and external stimuli (G_sn). (c) TRN thalamic input gains from the cortex (G_re), and SRN (G_rs). (d) Cortico-thalamo-cortical loop gains as a function of induced postsynaptic potential. As the system crosses the bifurcation point, both the TRN and SRN show a strengthened response to all inputs. In addition, the cortico-SRN-cortical loop gain, which resonates to given the characteristic alpha (~10 Hz) oscillation in wake, remains dominant.
