# Supplementary material for: Diffuse neural coupling mediates complex network dynamics through the formation of quasi-critical brain states
Source: Nat Commun. 2020 Dec 10;11:6337. doi: 10.1038/s41467-020-19716-7 (PMC7729877; doi:10.1038/s41467-020-19716-7)
Supplement: Supplementary file 3 — Reporting Summary [file 41467_2020_19716_MOESM3_ESM.pdf]

nature research

Corresponding author(s): Eli J. Muller

Last updated by author(s): 21/08/2020

Reporting Summary

Nature Research wishes to improve the reproducibility of the work that we publish. This form provides structure for consistency and transparency in reporting. For further information on Nature Research policies, see our Editorial Policies and the Editorial Policy Checklist.

Statistics

For all statistical analyses, confirm that the following items are present in the figure legend, table legend, main text, or Methods section.

n/a	Confirmed	
☐	☒	The exact sample size (*n*) for each experimental group/condition, given as a discrete number and unit of measurement
☒	☐	A statement on whether measurements were taken from distinct samples or whether the same sample was measured repeatedly
☐	☒	The statistical test(s) used AND whether they are one- or two-sided<br>*Only common tests should be described solely by name; describe more complex techniques in the Methods section.*
☒	☐	A description of all covariates tested
☒	☐	A description of any assumptions or corrections, such as tests of normality and adjustment for multiple comparisons
☐	☒	A full description of the statistical parameters including central tendency (e.g. means) or other basic estimates (e.g. regression coefficient) AND variation (e.g. standard deviation) or associated estimates of uncertainty (e.g. confidence intervals)
☐	☒	For null hypothesis testing, the test statistic (e.g. *F*, *t*, *r*) with confidence intervals, effect sizes, degrees of freedom and *P* value noted<br>*Give P values as exact values whenever suitable.*
☒	☐	For Bayesian analysis, information on the choice of priors and Markov chain Monte Carlo settings
☒	☐	For hierarchical and complex designs, identification of the appropriate level for tests and full reporting of outcomes
☐	☒	Estimates of effect sizes (e.g. Cohen's *d*, Pearson's *r*), indicating how they were calculated

Our web collection on statistics for biologists contains articles on many of the points above.

Software and code

Policy information about availability of computer code

Data collection	All numerical simulations were performed using nftSim v.1.1.0, a publicly available software package for simulating neural field models.
Data analysis	MATLAB R2018b was used for all analysis

For manuscripts utilizing custom algorithms or software that are central to the research but not yet described in published literature, software must be made available to editors and reviewers. We strongly encourage code deposition in a community repository (e.g. GitHub). See the Nature Research guidelines for submitting code & software for further information.

Data

Policy information about availability of data

All manuscripts must include a data availability statement. This statement should provide the following information, where applicable:

- Accession codes, unique identifiers, or web links for publicly available datasets
- A list of figures that have associated raw data
- A description of any restrictions on data availability

Human Connectome Project Database which is an openly available dataset

Field-specific reporting

Life sciences study design

All studies must disclose on these points even when the disclosure is negative.

Sample size	Sample size was the maximal available in the dataset
Data exclusions	No data was excluded from the analysis
Replication	The stochastic model fitting to the data was run ~1000 times
Randomization	Each run of the fitting algorithm contained a random weighting of different aspects within the data in order to prevent over-fitting.
Blinding	The analysis requires comparison between task and rest brain states, therefore blinding to group association was not appropriate.

Reporting for specific materials, systems and methods

We require information from authors about some types of materials, experimental systems and methods used in many studies. Here, indicate whether each material, system or method listed is relevant to your study. If you are not sure if a list item applies to your research, read the appropriate section before selecting a response.

Materials & experimental systems

n/a	Involved in the study	
☒	☐	Antibodies
☒	☐	Eukaryotic cell lines
☒	☐	Palaeontology and archaeology
☒	☐	Animals and other organisms
☐	☒	Human research participants
☒	☐	Clinical data
☒	☐	Dual use research of concern

Methods

n/a	Involved in the study	
☒	☐	ChIP-seq
☒	☐	Flow cytometry
☐	☒	MRI-based neuroimaging

Human research participants

Policy information about studies involving human research participants

Population characteristics	Please see Barch et al., 2014 (NeuroImage) for details.
Recruitment	Please see Barch et al., 2014 (NeuroImage) for details.
Ethics oversight	Please see Barch et al., 2014 (NeuroImage) for details.

Note that full information on the approval of the study protocol must also be provided in the manuscript.

Magnetic resonance imaging

Experimental design

Design type	task block/event design
Design specifications	Blocks varied across tasks (details are present in the methods section)
Behavioral performance measures	Responses were recorded using button presses. Please see Barch et al., 2014 (NeuroImage) for details.

Acquisition

Imaging type(s)	fMRI + diffusion MRI
Field strength	3T
Sequence & imaging parameters	3T<br>The following parameters were used for data acquisition: TR = 720 ms, echo time = 33.1 ms, multiband factor = 8, flip angle = 52 degrees, field of view = 208x180 mm (matrix = 104 x 90), 2x2x2 isotropic voxels with 72 slices, alternated LR/RL phase encoding.
Area of acquisition	Whole brain scan

Diffusion MRI ☒ Used ☐ Not used

Parameters: The spatial resolution was 1.25 mm isotropic, TR was 5500 ms, TE was 89.50 ms, the b-values were 1000, 2000, and 3000 s/mm2, and the total number of diffusion sampling directions was 90, 90, and 90 for each of the shells, in addition to 6 b0 images.

Preprocessing

Preprocessing software: Custom matlab scripts, which are available at github.com/macshine/

Normalization: Bias field correction and motion correction (12 linear DOF using FSL's FLIRT) were applied to the HCP resting state data as part of the minimal preprocessing pipeline54. To ensure equivalence across tasks, the data were also normalized within each temporal window, which effectively controlled for the global signal, while also equilibrating the data across independent subjects. Finally, a temporal low-pass filter (f < 0.125 Hz) was applied to the data

Normalization template: MNI152. Please see original study for details.

Noise and artifact removal: Temporal artifacts were identified in each dataset by calculating framewise displacement from the derivatives of the six rigid-body realignment parameters estimated during standard volume realignment55, as well as the root mean square change in BOLD signal from volume to volume (DVARS). Abnormal frames were not excluded from the data. However, we observed no significant relationship between any of the tPC time series and framewise displacement (estimated from the temporal head motion parameters) at the individual subject level (p > 0.5). Following artifact detection, nuisance covariates associated with the 12 linear head movement parameters (and their temporal derivatives), frame- wise displacement, DVARS, and anatomical masks from the CSF and deep cerebral WM were regressed from the data using the CompCor strategy56.

Volume censoring: Scrubbing was not performed on this data.

Statistical modeling & inference

Model type and settings: N/A

Effect(s) tested: Data was used for fitting to model ouputs

Specify type of analysis: ☐ Whole brain ☒ ROI-based ☐ Both

Anatomical location(s): Gordon et al. parcellation (cortex) + subcortical parcellation (Harvard/Oxford atlas) + cerebellar parcellation (SUIT atlas)

Statistic type for inference (See Eklund et al. 2016): N/A

Correction: N/A

Models & analysis

n/a	Involved in the study
☐	☒ Functional and/or effective connectivity
☐	☒ Graph analysis
☒	☐ Multivariate modeling or predictive analysis

Functional and/or effective connectivity: To estimate functional connectivity between the 375 ROIs, we used the Multiplication of Temporal Derivatives (M) technique59. M is computed by calculating the point-wise product of temporal derivative of pairwise time series (Equation 1). The resultant score is then averaged over a temporal window, w, in order to reduce the contamination of high-frequency noise in the time-resolved connectivity data. A window length of 20 TRs was used in this study, though results were consistent across a range of w values (10-50 TRs). To ensure relatively smooth transitions between each task, connectivity analyses were performed on each individual task separately, and were subsequently concatenated. In addition, all analyses involving connectivity (or the resultant topological estimates) incorporated the junction between each task as a nuisance regressor. Results were replicated using a jack-knife connectivity approach that does not require the fitting of a window.

Graph analysis: The Louvain modularity algorithm from the Brain Connectivity Toolbox (BCT60) was used in combination with the MTD to estimate time-resolved community structure. The Louvain algorithm iteratively maximizes the modularity statistic, Q, for different community assignments until the maximum possible score of Q has been obtained (see Equation 2). The modularity estimate for a given network is therefore a quantification of the extent to which the network may be subdivided into communities with stronger within-module than between-module connections.
